# The association between body image, emotional health, relationships, and unhealthy dietary behaviors among medical sciences students: A structural equation modeling analysis

**DOI:** 10.1002/npr2.12291

**Published:** 2022-09-07

**Authors:** Hoda Arabi‐Mianrood, Zohreh Shahhosseini, Monirolsadate Hosseini Tabaghdehi

**Affiliations:** ^1^ Department of Reproductive Health and Midwifery, Student Research Committee Mazandaran University of Medical Sciences Sari Iran; ^2^ Sexual and Reproductive Health Research Center Mazandaran University of Medical Sciences Sari Iran; ^3^ Department of Midwifery, Health Reproductive Research Center, Sari Branch Islamic Azad University Sari Iran

**Keywords:** body image, emotional health, relationships, unhealthy dietary behaviors

## Abstract

**Aim:**

Although it showed that poor body image may drive an individual to unhealthy dietary behaviors, but less is known about the role of emotional health and interpersonal relationships in this regard in different age group of population. This study aimed to investigate the association between body image, emotional health, relationships, and unhealthy dietary behaviors among medical sciences students.

**Methods:**

In this cross‐sectional study, using a three‐stage stratified sampling method, 248 Iranian medical sciences students were recruited. The participants completed self‐administered questionnaires, including Youth Risk Behavior Survey Questionnaire, Adolescent Health Concerns Inventory, and Body Image Concern Inventory. The data were analyzed using SPSS‐20 and structural equation modeling in the Amos software version 24.

**Results:**

The results indicate the acceptability of the goodness of fit model index. Direct association of body image with emotional health (*β* = 0.91, *p* = 0.005) and relationships (*β* = 0.88, *p* = 0.009) was significant, but any direct and indirect association between body and unhealthy dietary behaviors was not found.

**Conclusion:**

This study underlines the importance of being aware of the association between body image, emotional health, and relationships Further studies are recommended to explore the relationship between these factors and unhealthy dietary behaviors in medical sciences students.

## 1 INTRODUCTION

Unhealthy dietary behaviors are one of the health‐threatening issues that can lead to chronic diseases and mortality in the coming years of individuals' lives. These risky behaviors can be associated with upcoming poor health consequences such as diabetes, hypertension, as well as mental health problems including depression and anxiety.^1^, ^2^ The rate of unhealthy dietary behaviors is being increased during several past decades.^3^ The US Government has recommended healthy eating patterns for different age groups, including young people, which adjust calorie intake based on age, sex, height, weight, and physical activity.^4^

In this way it showed that over 40% of American students ignore government recommendations for healthy diet. Besides, approximately half of US adults are suffering from chronic diseases that are related to inadequate and low‐quality diet,^5^ as well as obesity has become a global phenomenon.^6^ However, this issue is varied among various cultures and ethnicities.^7^ Commitment to unhealthy dietary can be also influenced by several factors including sex, age, family and peers attitudes, and body image.^8^ So, it is worth noting that owing to the establishment of unhealthy dietary behaviors during youth ages, understanding the relationship between contributing factors to the dietary behaviors is vital for youth future health.^9^

Body image is a complex construction that comprises one’s thoughts, believes, and perception of their own body. Body schema is associated with several items such as gender, age, culture, marital status, psychopathology, education level, spirituality, and so on.^1^, ^10^ Various studies have shown that poor body image could have a great influence on physical and psychological health, accordingly can lead to anxiety, depression, lower self‐esteem, poor communication, social pressures, and so on. Consequently, these individuals are more likely to undertake various risky behaviors, such as unhealthy dietary behavior in order to relieve anxiety and worries about their appearance.^10^

By considering the relationship between body image and unhealthy dietary behaviors, it is assumed that it is a multidimensional and complicated concept, which needs to be addressed in order to establish a better understanding of youth dietary behaviors.^11^ Moreover, body schema can be influenced by youth emotional health and their personal and interpersonal relationships, including familial and societal relationships and feedbacks.^7^ Numerous studies in developed countries have shown that body image disorder is seen alongside unhealthy eating, which is on the rise in countries such as China, Malaysia, and Japan. Our understanding of this issue is limited due to a little evidence about the relationship between body image and dietary health‐threatening behaviors in non‐Western populations.^12^

Youngsters' body image could be affected by various social and environmental conditions. For instance, during college years, due to greater participation in the peer group, separation from family, being in more diverse social situations, they may be more likely to undertake unhealthy behaviors such as nutritional compensatory behaviors, dietary junk food to relieve psychological pressure.^13^, ^14^, ^15^ Owing to university life being considered as the transition period, when medical sciences students face some challenges in their relationships and social expectation of ideal body, whereas, they are expected to be part of the loop of health circulation.^16^

Given the importance of healthy dietary behaviors in medical students, the present study was designed to determine the path analysis fit of the conceptual model, which measures the mediating role of emotional health and relationships in the relationship between body image and unhealthy dietary behaviors among medical sciences students.

## 2 METHODS

### 2.1 Design and setting

This is a cross‐sectional study, which was conducted among 248 male and female medical sciences students of all six faculties of Mazandaran University of Medical Sciences (MAZUMS), North of Iran, from September to December 2018.

### 2.2 Sampling

Based on the correlation coefficient = 0.403, reported in a previous study in this issue,^17^ Alpha(type 1 error) = 0.05, Beta(type 2 error) = 0.02, and attrition rate = 15%, the sample size was calculated = 270. Participants were recruited by three‐stage stratified proportional to size sampling method. Initially, the sample size of each faculty of MAZUMS (including Dentistry, Medicine, Pharmacy, Public Health, Nursing and Midwifery, and Allied Medical Sciences) was calculated based on the total number of the students in that faculty divided by the total number of students in MAZUMS. Then, the number of samples allocated to each school was categorized based on the number of the male and female students in each school, alongside with their respective academic year. In turn, some classrooms in each school were selected randomly with the assistance of the random number table. Students, who attended in each class on the survey time, were selected by the random number table. Finally, they were invited to complete the anonymous self‐administered questionnaires.

### 2.3 Inclusion and exclusion criteria

Medical sciences students aged between 18 and 25, who studied for a Bachelor’s degree in Science or General Physician, were enrolled. Postgraduate students were not included in the study due to their different concerns as well as their older age than undergraduate students.

### 2.4 Measurements

#### 2.4.1 Demographic information

It included some questions about participants' characteristics such as age, gender, marital status, body mass index, field of study, and their parents' education and job.

#### 2.4.2 Youth risk behavior survey questionnaire

To assess participants' dietary behaviors, seven relevant questions from this questionnaire were adapted. The dietary items included the following: during the 7 days before the survey, had eaten fruit or drunk 100% fruit juices, had eaten vegetables (lettuce, tomato, or other vegetables), had eaten breakfast, had drunk canned or carbonated drinks, and had drunk a few glasses of milk. In each question, the number of times of each dietary behavior was reported from zero time/day to ≥3 times/day (except for dietary breakfast from zero to seven times). The more times an individual reported each dietary behavior implies the less unhealthy behavior (except for drinking canned or carbonated drinks which its score was reverse). The first version of this questionnaire was developed in 1997 by the Centers for Disease Control and Prevention by an acceptable reliability of 60.7 (kappas ranged from 23.6% to 90.5% with a mean of 60.7%).^18^ The psychometric properties of this questionnaire in Iranian setting were established by Cronbach alpha = 0.97 and mean ICC for domain body weight and dietary behavior was 0.85.^13^

#### 2.4.3 Body image concern inventory

It was designed by Littelton et al. to assess dysmorphic concerns, in 2005. The inventory measures the extent to which individuals are satisfied with their body image, contained 19 questions (including 12 and seven questions about dysmorphic and interference symptoms, accordingly), on a five‐point Likert scale ranging from 1 = (never) to 5 = (always). Scores entire possible range is 19–95. The total score of the questionnaire was determined by calculating the mean total score. In this questionnaire, the more the total scores, the more positive body image. Its Cronbach alpha coefficient and its internal validity coefficient were reported as 0.93 and 0.92, respectively. Its reliability was established by Cronbach alpha coefficient for Iranian female and male students as 0.93 and 0.95 accordingly.^19^

#### 2.4.4 Adolescent health concerns inventory

To assess participants' emotional health and their relationships, this inventory was used. The emotional health (17 items) refers to items such as: having a psychological balance, being anxious, dying, loneliness, feeling guilty, feeling good about one’s self, being compared with others, and so on. The relationships included 14 items such as: getting alone with parents, siblings, friends, getting married, falling in love, having a boyfriend or girlfriend, and so on. This inventory was developed by Weiler in 1990 and each item is scored as either 0 or 1, and the higher scores indicated poor emotional health and poor interpersonal relationships.^20^ The internal consistency of the Persian version of this inventory was determined by Cronbach alpha coefficient 0.96 and the Kappa coefficients 0.75, which demonstrate an acceptable compromise of the inventory.^21^

### 2.5 Data analysis

The data were then analyzed using SPSS‐20. In describing numerical variables, mean and standard deviation were used and to describe categorical variables, frequency and percentage were used. The main variables studied in the model were unhealthy dietary behavior as the dependent variable, realationships and emotional, health as the mediating variables, and body image, concern, invertory as the independent variables. The relationship between the main variables of the research was performed using Amos software version 24. The main basis of data analysis was on Structural Equation Modeling. Significance level in all tests was considered 0.05.

### 2.6 Ethical considerations

Ethical code from Mazandaran University of Medical Sciences (Ethical code: IR.MAZUMS. REC. 95.2446) and written informed consent from the volunteer participants were of ethical considerations. Explaining the research project objectives for participants and assurance about the secrecy of the collected data were the others.

## 3 RESULT

From 270 questionnaires, data from 22 ones were not included to the final analysis due to incomplete data (Response rate = 91.8%). The mean age of participants was 21.40 ± 2.02 and 53.6% of them were male. Most of the participants (59.5%) were studying for a Bachelor’s degree and (39.5%) General Physician students in Science. Most of them (90.3%) were single, and 45.9% lived in dormitories. Table 1 shows 177(71.4%) of the participants had eaten breakfast <7 days during the 7 days before the enrollment, 73(29.4%) had drunk a few glasses of milk <1 time/day, and had eaten canned or carbonated drinks ≥1 time/day at the same period. Other characteristics of the participants are shown in Table 1.

**TABLE 1:** Characteristics of medical sciences students (*n* = 248)

Variables	Frequency	Percentage
Age (Mean ± SD) 21.40 ± 2.02	–	–
BMI (Mean ± SD) 23.07 ± 4.10	–	–
Gender		
Male	133	53.6
Female	115	46.4
Grade		
Bachelor	148	59.5
Doctorate	100	40.5
Marital status		
Married	22	8.9
Single	223	90.3
Divorced	3	0.8
Residency		
Urban	206	83
Rural	42	17
Drank 100% fruit juices		
<1 time/day	144	58.1
≥1 time/day	104	41.9
Ate fruit		
<1 time/day	26	10.5
≥1 time/day	222	89.5
Ate vegetables such as lettuce, tomato		
<1 time/day	45	47.6
≥1 time/day	203	52.4
Ate other vegetables		
<1 time/day	118	47.6
≥1 time/day	130	52.5
Ate canned or carbonated drinks		
<1 time/day	78	31.5
≥1 time/day	170	68.5
Drank a few glasses of milk		
<1 time/day	73	29.4
≥1 time/day	175	70.6
Ate breakfast		
<7 days	177	71.4
≥7 days	71	28.6

As shown in Table 2, Cronbach alpha is above 0.7 for all variables in this study that indicates the appropriateness of the questionnaire for research purposes. Results of Pearson correlation coefficient showed that emotional health (*r* = −0.16*, p* < 0.001) and relationships (*r* = −0.19, *p* < 0.001) are adversely correlated with unhealthy dietary behaviors (Table 3).

**TABLE 2:** Mean, standard deviation, and Cronbach alpha of variables

Variables	Items (range of scores)	Mean	Standard deviation	Cronbach alpha
Unhealthy dietary behaviors	7 questions (7–35)	11.58	0.08	0.91
Emotional health	17 questions (0–17)	4.22	0.28	0.91
Relationships	14 questions (0–14)	3.79	0.23	0.91
Body image concern	Dysmorphic symptoms‐ 12 questions (12–60)	25.71	0.55	0.91
	Interference symptoms‐ 7 questions (7–35)	11.51	0.55	0.91

**TABLE 3:** Correlation analysis between variables

Variables	1	2	3	4	5
1. Relationships	1				
2. Emotional health	0.72*	1			
3. Body image concern‐dysmorphic symptoms	0.24*	0.26*	1		
4. Body image concern‐ interference symptoms	0.27*	0.30*	0.68*	1	
5. Unhealthy dietary behaviors	−0.19*	−0.16*	−0.10	−0.07	1

*p < 0.001.

**FIGURE 1:**
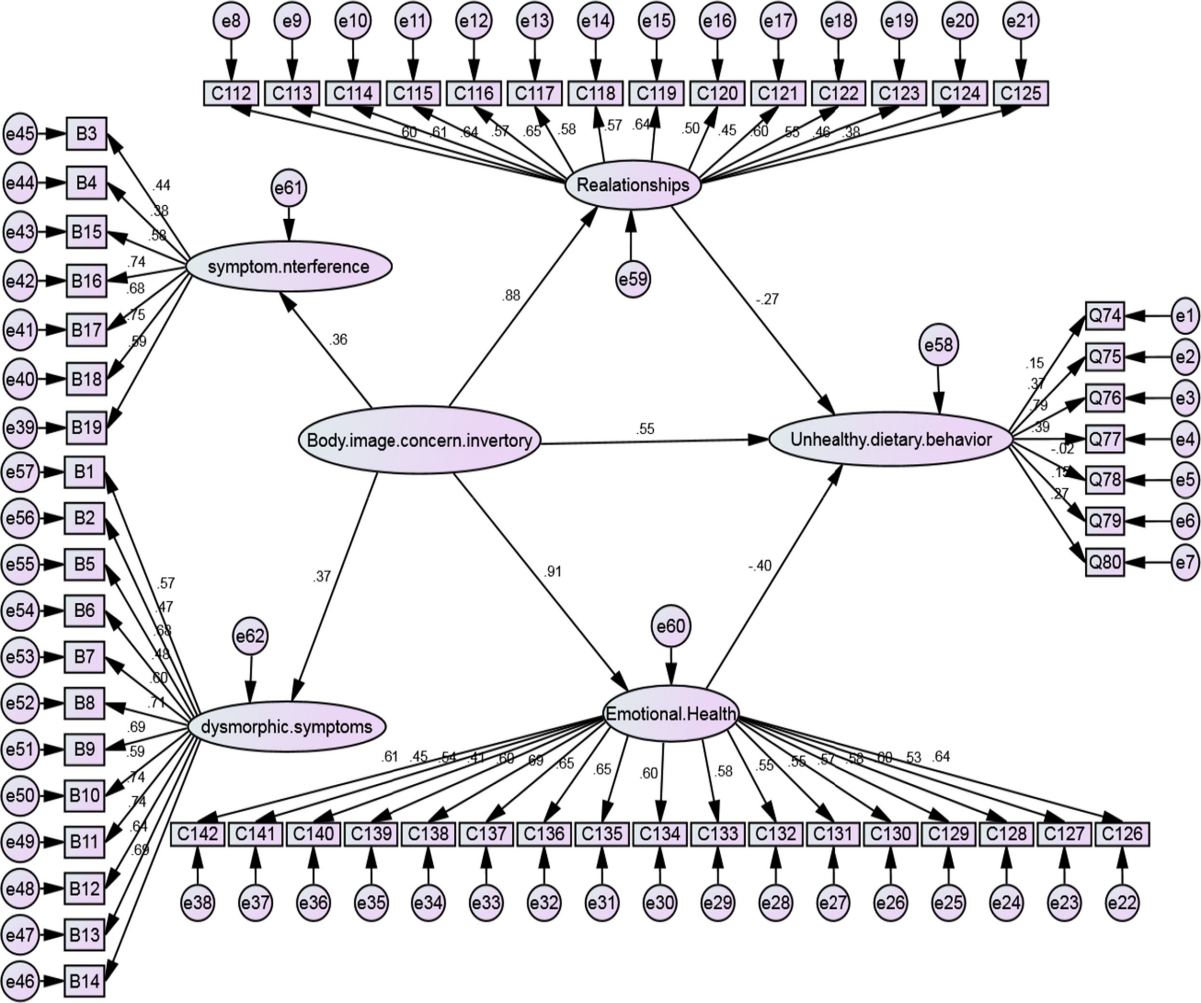
The final model of research and its parameters were investigated by structural equations modeling using Amos 24.0 software and the maximum likelihood. The χ^2^/*df* = 1.90, the comparative fit index (CFI) = 0.88, the parsimony comparative fit index (PCFI) = 0.65, and the root mean square error of approximation (RMSEA) = 0.06. Direct association of body image with emotional health (*β* = 0.91) and relationships (*β* = 0.88) was significant, but any direct and indirect association between body and unhealthy dietary behaviors was not found

In this study, mean ± SD emotional health and relationship were 4.22 ± 0.28, 3.79 ± 0.23 respectively. The final model of research and its parameters were investigated by structural equations modeling using Amos 24.0 software and the maximum likelihood (Figure 1). In this way, the χ^2^/*df* = 1.90, the comparative fit index (CFI) = 0.88, the parsimony comparative fit index (PCFI) = 0.65, and the root mean square error of approximation (RMSEA) =0.06 indicating the acceptability of the goodness of fit model index.^22^

As reported in Table 4, the direct and indirect associations of body image concern on unhealthy dietary behavior were investigated. Although the estimated direct (*β* = 0.55) and indirect (*β* = −0.61) associations were relatively high numerically, their relationships were not statistically significant (*p* = 0.846). Results showed that body image concern could have effect on emotional health (*β* = 0.91, *p* = 0.005) and relationships (*β* = 0.88, *p* = 0.009) directly.

**TABLE 4:** Results for standardized effects of variables

Variables	Total effects	Direct effect	Indirect effect	Lower bounds	Upper bounds	T‐value	*p*‐value
Body Image concern → Emotional health	0.91	0.91	–	0.83	1.01	4.00	0.005
Body Image concern → Relationships	0.88	0.88	–	0.78	0.97	4.01	0.009
Body Image concern → Dysmorphic symptoms	0.37	0.37	–	0.21	0.50	3.35	0.016
Body Image concern → Interference symptom	0.36	0.36	–	0.13	0.53		0.014
Body Image concern → Unhealthy dietary behavior	−0.06	0.55	−0.61	−0.35	0.27	0.38	0.846
Emotional health→ Unhealthy dietary behaviors	−0.40	−0.40	–	−2.02	2.32	−0.47	0.730
Relationships→ Unhealthy dietary behaviors	−0.27	−0.27	–	−1.86	1.10	−0.43	0.783

## 4 DISCUSSION

The aim of the study was: “This study aimed to investigate the association between body image, emotional health, relationships, and unhealthy dietary behaviors among medical sciences students.” This is one of the limited studies conducted on this issue in a developing country that may be a beginning for future studies and broaden our understanding in this regard.

The finding of the present study about nonsignificant relationship between body image and unhealthy dietary behaviors maybe as a result of the complexity of the related factors to an individual’s dietary behaviors such as the psycho‐cultural context, personality, interpersonal relationships, dietary preferences, health values, peers pressure, and stressors.^2^, ^23^, ^24^

A considerable number of studies show that poor body image directly increases the vulnerability to unhealthy diet behaviors, for example, it is indicated that feeling does not have desired body, increases the odds of fasting or any unhealthy dietary behaviors.^25^ Also, it is reported when individuals find their own body differs from the ideal society body scheme, they may feel down and lack self‐esteem. Subsequently, they may undertake unhealthy dietary behaviors, in order to lose weight.^26^ Contrary, individuals with greater positive body image and higher self‐esteem reported less depression, a higher rate of healthy diet, and were more intended to protect their body.^27^, ^28^ Different from the results of above studies, a review by Duarte et al. found limited evidences about direct association between body satisfaction and risky dietary behaviors such as lack of vegetables and fruits consumption and drinking sodas.^29^ Also, it is reported that relationships with parents, peer groups, and society can affect young people beliefs and inspire them about the ideal body, so they are more likely to commit healthy dietary behaviors in order to achieve the desired body.^12^

In this study, it was shown that better body image of medical sciences students may lead to their better emotional health and better relationships. Satisfaction with body image as a emotional component has an effective role in internalizing the mental health of adolescents.^30^ Individuals with a high level of positive body image are less prone to depression and have high self‐esteem, and such individuals love and respect their physical self and also pay more attention to their emotional health, and as a result, take more self‐care behaviors.^27^

Also in this regard, it showed that favorite adolescents' relationships with their parents reduce body dissatisfaction and lead to a positive body image.^1^ Proper communication with family members has a positive effect on body image. Positive body image promotes emotional health by increasing self‐esteem. So that in the surveys it was shown that expressing critical opinions, mocking, and encouraging the diet by the family play a significant role in the dissatisfaction of adolescents.^6^ There have been several studies on parents and peer relationships on body image,^31^, ^32^. but no study has been done on the body image effect on parents and peer relationships, so it is suggested that more studies be done in different age groups.

### 4.1 .Strengths and imitations

The structural equation modeling analysis as a multivariate statistical analysis technique was used to analyze the structural relationship between measured variables and latent constructs related to unhealthy dietary behaviors. Also, as entering the Iranian university is followed by a national entrance exam (named KOONKOOR), so the participants were students from all over the country and from different ethnic groups. This could increase the generalizability of the findings. Our findings must be interpreted in the light of some limitations. Due to the cross‐sectional design of this study, it is impossible to determine the cause‐and‐effect relationships between variables because of temporality bias. Second, information bias is possible due to self‐reported data gathering technique. As a result of culturally sensitive questions such as relationships with parents and opposite gender, especially in conservative societies such as Iran, social desirability bias is anticipated. To reduce this bias, the participants were assured about the confidentiality of the information. Finally, the results may not be generalized to non‐medical sciences students. It is suggested to conduct a similar study in non‐medical students in different academic levels.

## 5 CONCLUSION

This study underlines the importance of being aware of the relationships between body image, emotional health, and relationships. Medical sciences students with poor body image should be assessed for their emotional health and their relationships. Also, as this is a cross‐sectional study, body image concern of individuals with poor emotional health and weak relationships must be investigated. Further studies are recommended to explore the relationship between these factors and dietary behaviors.

## AUTHOR CONTRIBUTIONS

Hoda Arabi‐Mianrood and Monirolsadate Hosseini Tabaghdehi contributed to the design of the study. Hoda Arabi‐Mianrood, Zohreh Shahhosseini, and Monirolsadate Hosseini Tabaghdehi contributed to the implementation and analysis plan. Hoda Arabi‐Mianrood contributed to data collection. Hoda Arabi‐Mianrood, Monirolsadate Hosseini Tabaghdehi, and Zohreh Shahhosseini have written the first draft of this manuscript.

## CONFLICT OF INTEREST

No conflict of interest.

## APPROVAL OF THE RESEARCH PROTOCOL BY AN INSTITUTIONAL REVIEWER BOARD

N/A.

## REGISTRY AND THE REGISTRATION NO. OF THE STUDY/TRIAL

N/A.

## ANIMAL STUDIES

N/A.

## Data Availability

Since our data contain sensitive personal information, it is forbidden to share these data with a third party without obtaining an additional written form of informed consent for information sharing. We did not obtain the additional written consent for information sharing.
